# Physician’s Diary

**Published:** 1856-09

**Authors:** 


*Physician's Diary.*—At the request of several parties we have prepared a form for a Physician's Diary, which will be published by Phinney & Co., of this city, in time for the fall trade. We have attempted to combine a convenient size for daily use in the pocket, with sufficient space for the record of some of the essential particulars of disease. We had hoped that the form published last year, by Phinney & Co., would have been satisfactory, but serious objections were found to its use. It was too large and weighty to carry comfortably in the pocket, and too much was attempted, both in the medical and obstetrical record. Again, the medical record was at the beginning of the book, it always required a search to find the visiting list, and the separation of the two from each other caused the record to be entirely neglected, even had the spaces assigned to the record of facts been large enough to be useful.

The size of the "Physician's Diary" is just that of the well-known "Visiting List," published by Lindsay & Blakiston, and only one-half as large as the other form alluded to. It contains a page for each week, allowing the record of thirty patients, while *directly on the opposite page* is ample space for the age of the patient, the name of the disease, the termination of the case, and remarks. By this plan the name of the patient need be written but once, and there is a constant *temptation* to fill out the line and complete the record of the case. It is not intended that the "Diary" should take the place of the case-book, but if the spaces are all filled out, it does record the the name of the patient, sex, time of first medical visit, duration and amount of attendance, time of last visit, age, diagnosis, termination, and any one or two other facts as to character of disease, treatment, or locality, which may be deemed most important.

The obstetrical record occupies but eight pages, affording space for 240 cases. It gives the name, age, number of pregnancy, presentation, sex of child, hours in labor, and character of labor, whether natural or artificial. It must be recollected that the space for the record of these facts is *ample.* The date of labor must be given by the visiting list. There are also a few pages devoted to "Nurses Addresses," "List of Things Lent," and "Bills to be Presented." In the opening of the book is given a table of signs, a list of poisons and antidotes, a dose table, almanac, preface, etc.

The "Diary" will be sold at 75c. retail price. Orders may be addressed, enclosing money or stamps, to the publishers, or to the address of this Journal. The binding will be permanent and strong. The mode of fastening will be by India-rubber straps, which are much better than the usual "tuck."

